# A PhoP-regulated type II secretion system drives bacterial competition in *Serratia marcescens*

**DOI:** 10.1128/spectrum.02169-26

**Published:** 2026-10-15

**Authors:** María Sol Sartori, Brayan S. Arango Gil, Eleonora García Véscovi, Javier F. Mariscotti

**Affiliations:** 1 Instituto de Biología Molecular y Celular de Rosario, Consejo Nacional de Investigaciones Científicas y Técnicas, Universidad Nacional de Rosario 63031, Rosario, Argentina; Universidad Maimonides, Buenos Aires, Argentina

**Keywords:** *Serratia*, PhoP/PhoQ, type II secretion system, bacterial competition

## Abstract

*Serratia marcescens* is an enteric bacterium that can act as an opportunistic pathogen, with increasing incidence in clinical settings. This is largely due to its ability to express a broad repertoire of virulence factors and acquire antibiotic resistance mechanisms. Consequently, *S. marcescens* has been designated by the World Health Organization (WHO) as a research priority for the development of alternative antimicrobial strategies. In this study, we identify a previously unrecognized role of the type II secretion system (T2SS) in mediating bacterial competition in the clinical strain *S. marcescens* RM66262. Phylogenomic analysis reveals that the T2SS is conserved among clinical isolates but absent from most environmental strains, suggesting it confers a selective advantage in host-associated or highly competitive environments. Functional assays demonstrate that the T2SS contributes to both inter- and intraspecies antagonism and acts in concert with the contact-dependent type VI secretion system (T6SS), with both systems contributing additively to bacterial killing through distinct mechanisms. We further show that T2SS expression is positively regulated by the PhoP/PhoQ two-component system through direct binding to its promoter region and is induced in response to competitive cues such as attack by *Acinetobacter nosocomialis*. Altogether, our findings expand the functional scope of the T2SS beyond classical extracellular virulence, establishing it as a PhoP-regulated system involved in bacterial competition and a promising target for novel anti-virulence strategies.

**IMPORTANCE:**

This work uncovers a previously uncharacterized role for the type II secretion system (T2SS) in *Serratia marcescens*, demonstrating its involvement in both inter- and intraspecies bacterial competition. The finding that the T2SS is conserved among clinical isolates but largely absent in environmental strains suggests that this system confers a selective advantage in host-associated or high-competition settings. Furthermore, we show that T2SS expression is regulated by the PhoP/PhoQ system and induced by interbacterial contact, underscoring its role in adaptive antagonistic responses. By expanding the known functional scope of the T2SS, this study provides new insights into the ecological strategies of opportunistic pathogens and highlights a promising target for anti-virulence therapeutic development.

## INTRODUCTION

*Serratia marcescens* is recognized as an opportunistic human pathogen with a growing clinical incidence, particularly in hospitalized and immunocompromised individuals. This bacterium is associated with a wide range of infections, including those of the urinary and respiratory tracts, as well as wound and eye infections, endocarditis, osteomyelitis, meningitis, and septicemia. The increasing prevalence of *S. marcescens* infections is often linked to the acquisition of multiple antibiotic resistances, posing a significant challenge in clinical settings (1–4). In fact, *S. marcescens* has been identified by the World Health Organization (WHO) as a research priority for the development of alternative antimicrobial strategies due to its increasing resistance to antibiotics (5). In recent years, outbreaks of multidrug-resistant strains and high incidence rates in intensive and neonatal care units have been increasingly reported (6–8). Moreover, *S. marcescens* has been identified as one of the three most abundant microbial species colonizing the dysbiotic gut of patients with Crohn’s disease (9). Beyond human infection, *S. marcescens* can establish both symbiotic and pathogenic interactions with plants and insects, and its ability to produce a wide array of extracellular enzymes and secondary metabolites enables it to thrive in diverse and dynamic environments (10). These features underscore the remarkable ecological plasticity of *S. marcescens* and its capacity to adapt to complex polymicrobial niches.

In this study, we identified the presence of a type II secretion system (T2SS) in the clinical strain RM66262 (11), as well as in multiple clinical isolates of *S. marcescens*, whereas it was largely absent from environmental strains. This observation is consistent with the work of Iguchi et al. (12), who first reported the presence of the T2SS in a clinical *S. marcescens* isolate, whereas the environmental isolate analyzed in the same study lacked this secretion system. A recent population genomic study encompassing more than 200 clinical and environmental *S. marcescens* isolates, including RM66262, independently revealed a strong association between T2SS genes and hospital-adapted or clinically prevalent lineages, further supporting the distribution pattern observed in our analysis (13). Consistently, additional genomic studies have delineated well-defined lineages predominantly associated with clinical settings, and RM66262 clusters within these lineages (14, 15). Together, these findings reveal a marked separation between clinical and environmental *S. marcescens* lineages and show that the T2SS is predominantly associated with clinically relevant strains, suggesting that this secretion system contributes to adaptation, competitive fitness, and persistence in host-associated environments. Although the substrates of the *S. marcescens* T2SS remain unidentified, its preferential association with clinically relevant lineages points to a previously underappreciated role in virulence and interbacterial interactions.

The T2SS is a sophisticated multiprotein machinery employed by many gram-negative bacteria to transport folded proteins across the outer membrane into the extracellular milieu or onto the cell surface. It operates in a two-step process in which substrates are first translocated across the inner membrane via the Sec or Tat pathways and subsequently secreted through the outer membrane by the dedicated T2SS apparatus. This system typically comprises 12–15 proteins, of which 12 core components (GspCDEFGHIJKLMO) are essential for full functionality (16). T2SS substrates are diverse and include numerous virulence factors, such as toxins and hydrolytic enzymes (proteases, lipases, and chitinases), which contribute to processes such as pathogenesis, nutrient acquisition, and biofilm formation in a wide range of gram-negative bacteria (16–21). However, recent studies have expanded this paradigm, demonstrating that the T2SS also mediates interbacterial antagonism by delivering antibacterial effectors—particularly peptidoglycan-degrading enzymes—that eliminate competing bacteria across diverse ecological niches (22). Accordingly, the T2SS is not restricted to host-associated virulence or nutrient acquisition but also represents a key determinant of bacterial competition and survival in both clinical and environmental contexts. Despite the broad functional diversity of T2SSs described in other gram-negative bacteria, the biological role of this secretion system in *S. marcescens* has remained completely unexplored. In particular, neither its contribution to virulence nor its potential involvement in interbacterial interactions has been previously investigated.

Here, we investigated the role of the T2SS in the virulence of *S. marcescens* RM66262. We show that this system contributes to both inter- and intrabacterial competition and is transcriptionally regulated by the PhoP/PhoQ two-component system. Although transcriptional regulation of T2SSs has been described in other bacteria, including the Yts1 system of *Yersinia enterocolitica*, where expression is controlled by environmental signals and global regulators (23), regulation by the PhoP/PhoQ two-component system has not been reported. Thus, to our knowledge, this study represents the first evidence linking PhoP/PhoQ to the regulation of T2SS expression in *S. marcescens* or any other bacterial species. Furthermore, PhoP-dependent modulation of bacterial killing is triggered upon contact with competitor cells. Notably, the T2SS is preferentially associated with clinically relevant lineages, supporting its role in adaptation to host-associated or high-competition niches. Moreover, this study provides the first evidence that the *S. marcescens* T2SS contributes to bacterial competition. Together, our findings establish the T2SS as a PhoP-controlled determinant of competitive fitness and reveal a previously unrecognized strategy employed by *Serratia* to thrive in polymicrobial environments.

## RESULTS

### Genomic analysis and phylogenetic relationship of the T2SS in clinical and environmental isolates of *Serratia marcescens*

**Fig 1:**
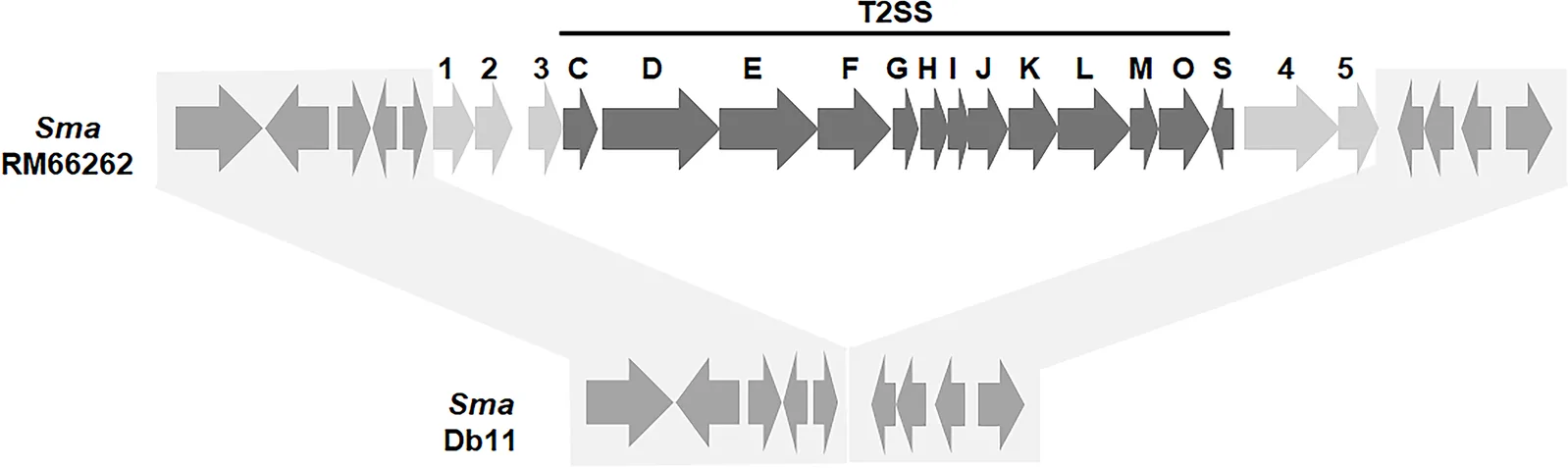
Type II secretion system of *S. marcescens* RM66262. Schematic representation of the organization of the T2SS genes of *S. marcescens* RM66262 compared to *S. marcescens* Db11. 1: RT90_RS11385; 2: RT90_RS11380; 3: RT90_RS11375; C: *gspC*; D: *gspD*; E: *gspE*; F: *gspF*; G: *gspG*; H: *gspH*; I: *gspI*; J: *gspJ*; K: *gspK*; L: *gspL*; M: *gspM*; O: *gspO*; S: *gspS*; 4: *gbpA*; 5: RT90_RS11300.

The genome of the clinical isolate *S. marcescens* RM66262, sequenced in our laboratory, was compared to that of the environmental strain *S. marcescens* Db11 through an *in silico* analysis, focusing on the gene cluster encoding the type II secretion system (T2SS). A comparative genomic analysis revealed that the clinical isolate possesses an 18-gene region absent in *S. marcescens* Db11, with 13 of these genes encoding structural components of the T2SS. According to predictions generated using the FGENESB algorithm (24), all structural genes of the T2SS—except gene *S*—along with the upstream gene *RT90_RS11375* (located upstream of gene *C*), are likely part of the same operon (Fig. 1).

**Fig 2:**
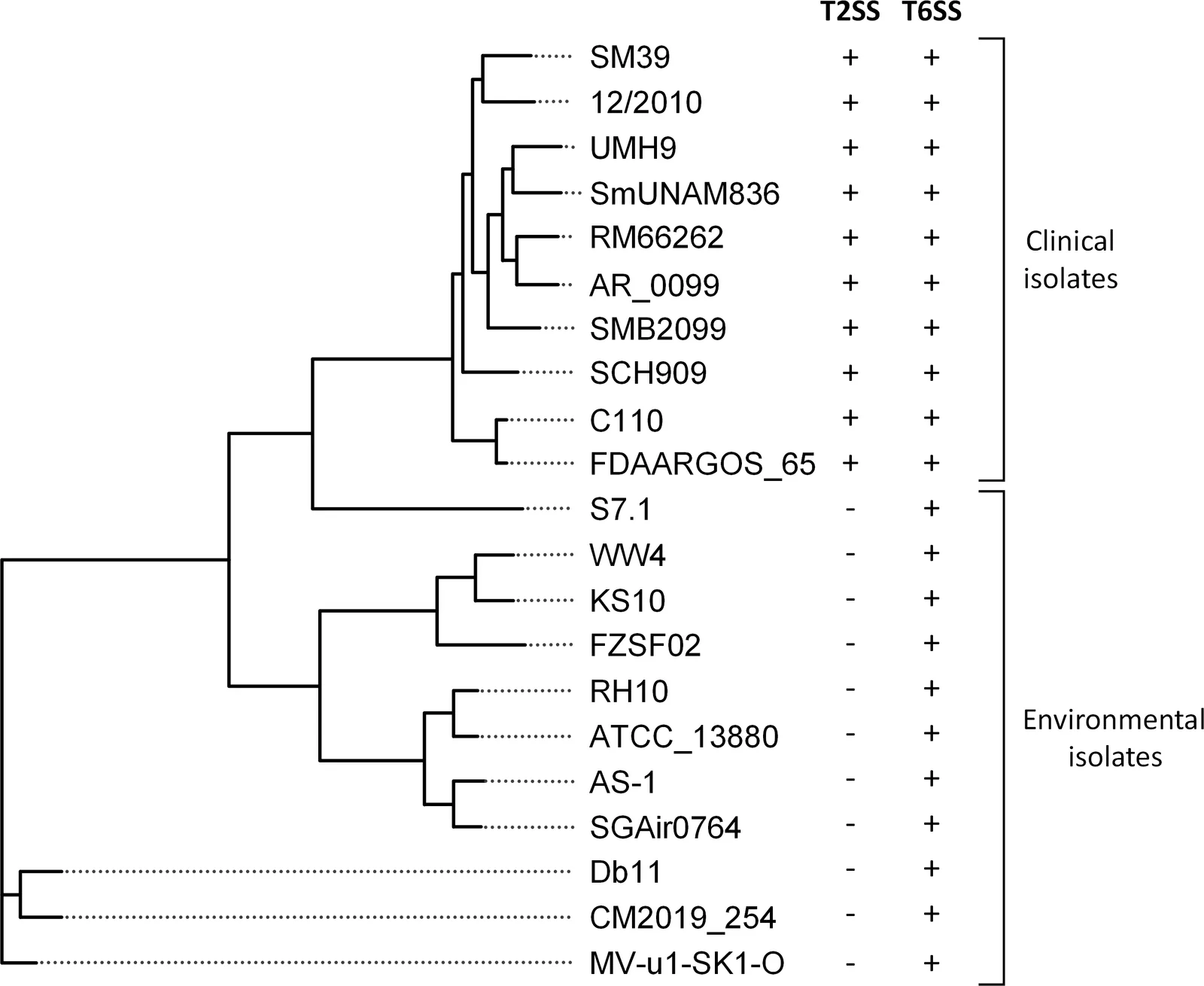
Phylogenetic tree based on the complete chromosome sequences of 21 strains of *S. marcescens*. The phylogenetic tree was constructed by REALPHY 1.13. The presence of the T6SS and T2SS was indicated on the right side of the tree.

To verify the presence of the T2SS in *S. marcescens* strains and elucidate its evolutionary relationships, we constructed a phylogenetic tree using the complete chromosomal sequences of 21 *S. marcescens* strains, including 10 clinical and 11 environmental isolates. The tree was generated using the REALPHY 1.13 server (25), and the presence or absence of T2SS-encoding genes was subsequently assessed (Fig. 2). Our analysis revealed that the T2SS is consistently present in all clinical isolates but absent from the environmental strains analyzed. In contrast, the type VI secretion system (T6SS) was detected in all strains regardless of origin. Notably, T2SS-positive clinical isolates clustered together, forming a distinct phylogenetic group clearly separated from environmental isolates lacking the system (Fig. 2).

These findings are consistent with recent population genomic studies, such as those reported by Ono et al. (13), which identified the T2SS as a feature enriched in hospital-adapted lineages of *S. marcescens*. Together, our results reinforce the notion that the T2SS is associated with clinically relevant strains and may represent a lineage-specific trait linked to adaptation to host-associated or high-competition environments.

### The T2SS is involved in bacterial competition

Previous studies have demonstrated that the type VI secretion system (T6SS) of *S. marcescens* plays a role in bacterial competition (26–28). However, the involvement of the T2SS in this process has not been described. In this work, we investigated whether the T2SS also contributes to bacterial antagonism in *S. marcescens*.

To address this question, we performed bacterial competition assays using *Escherichia coli* MC4100 as prey and *S. marcescens* RM66262 as attacker, including both wild-type (WT) and T2SS-deficient mutant (*gspD*) strains. GspD is the key outer membrane component of the T2SS, and its correct assembly and localization are essential for the proper function of the entire secretion machinery (29–31). Additionally, we included a *tssM* mutant defective in the T6SS, which has been previously implicated in bacterial competition (28). As a control, we used *E. coli* DH5α as the attacker; this laboratory strain, like MC4100, lacks a T6SS in its genome (28).

**Fig 3:**
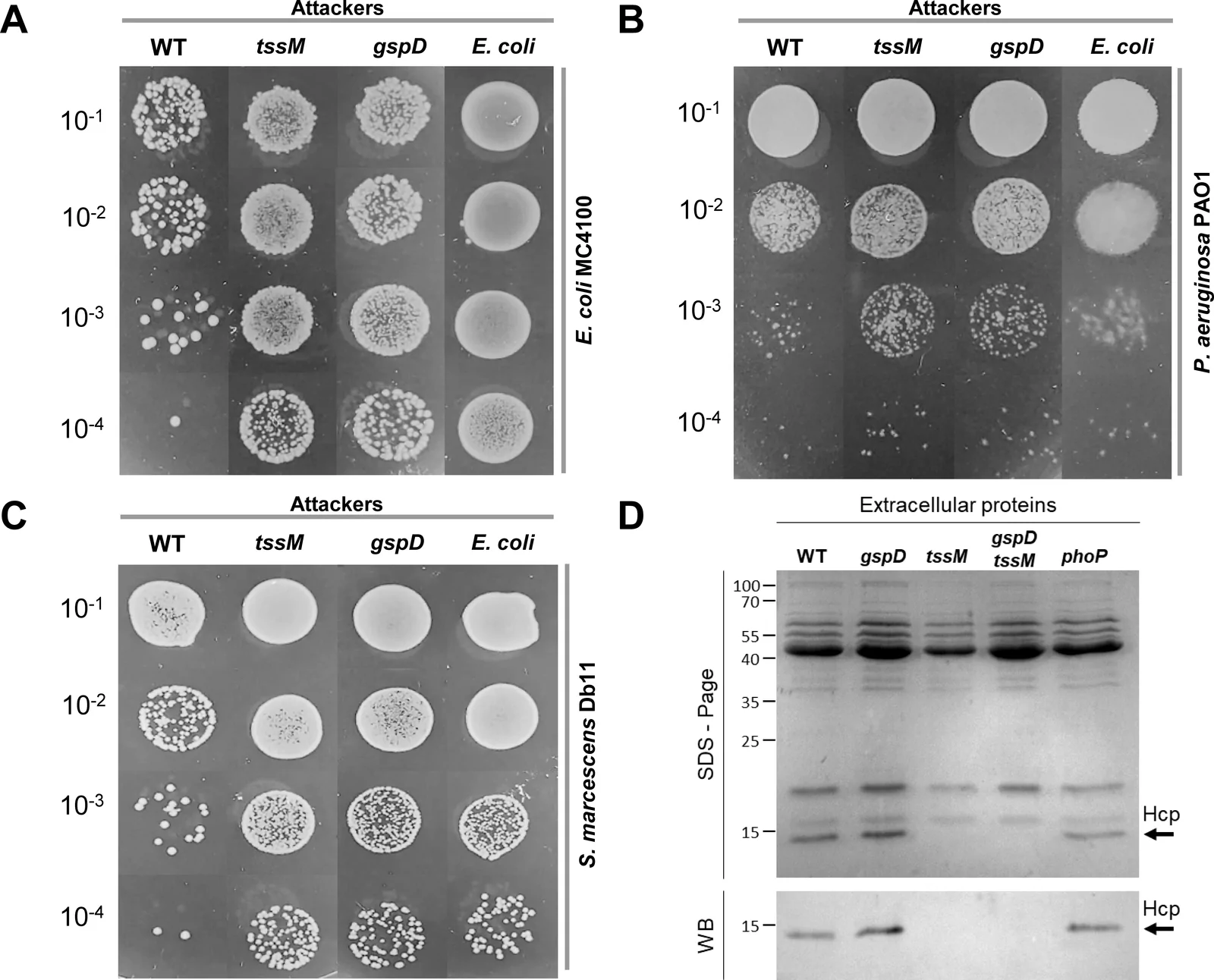
Analysis of T2SS functionality by interbacterial competition assays. Wild-type (WT), T6SS mutant (*tssM*), and T2SS mutant (*gspD*) *S. marcescens* RM66262 were used as attackers. *E. coli* MC4100, *P. aeruginosa* PAO1, or *S. marcescens* Db11 were used as prey, as indicated for each assay. Strains were co-incubated in a 5:1 or 10:1 ratio (attacker:prey) at 37°C for 4 h. *E. coli* DH5α was co-incubated with the prey as a control. The prey strain was selected by plating on the corresponding antibiotic. Representative images of three independent assays are shown in the figure. (**A**) *S. marcescens* RM66262 vs *E. coli* MC4100 using a 5:1 ratio. (**B**) *S. marcescens* RM66262 vs *P. aeruginosa* PAO1 using a 10:1 ratio. (**C**) *S. marcescens* RM66262 vs *S. marcescens* Db11 using a 5:1 ratio. (**D**) Analysis of the presence of T6SS-secreted Hcp. Protein precipitation was performed with the supernatants of the WT, *gspD*, *tssM*, *gspD tssM,* and *phoP* strains. Hcp levels were determined by immunodetection using Hcp antiserum. The figure shows a representative image of two independent assays.

Liquid co-cultures of the attacker and prey were preincubated at a 5:1 ratio and subsequently spotted on Luria-Bertani (LB)–agar in 24-well plates. After incubation at 37°C for 4 h, the bacterial mixtures were resuspended in LB, serially diluted in phosphate-buffered saline (PBS), and plated on LB–agar supplemented with streptomycin to select for the prey. The results showed that inactivation of the T2SS significantly reduced the ability of *S. marcescens* to eliminate the prey strain, similar to what was observed for the T6SS mutant (Fig. 3A). To rule out the possibility that the reduced competitive ability resulted from a general growth defect, we compared the colony-forming unit (CFU) counts of the attacker strains under the same conditions used for the competition assays. All mutant strains exhibited growth comparable to that of the WT, with no significant differences in final CFU counts, indicating that the impaired competitive phenotypes were not attributable to general growth defects (See Fig. S1 at https://ibr-conicet.gov.ar/wp-content/uploads/2026/09/Spectrum-Sartori-et-al-Supplemental-Material.pdf).

Given that *S. marcescens* RM66262 can eliminate *E. coli*, we investigated whether this strain could also target other species or even other *S. marcescens* strains in a T2SS-dependent manner. The competition assay was performed using either *Pseudomonas aeruginosa* (Fig. 3B) or *S. marcescens* Db11 (Fig. 3C) as prey. In both cases, results were similar to those obtained when *E. coli* was used as prey, confirming that the T2SS endows *S. marcescens* with interspecies and intraspecies competition capacity.

Since T2SS-deficient mutants exhibited a competition-defective phenotype similar to that of the T6SS mutant, we evaluated whether T2SS-deficient mutants retained a functional T6SS. The presence of Hcp, a hallmark of active T6SS assembly, was assessed in the culture supernatants of *S. marcescens* WT, *gspD* (T2SS mutant), *tssM* (T6SS mutant), *gspD tssM* (double mutant), and *phoP* strains. Secreted proteins were analyzed by sodium dodecyl sulfate polyacrylamide electrophoresis (SDS-PAGE), followed by Coomassie blue staining or immunodetection using an anti-Hcp antibody. The results showed the presence of Hcp in the *gspD* strain, consistent with the absence of interference with T6SS assembly following T2SS inactivation (Fig. 3D).

In conclusion, our findings demonstrate that *S. marcescens* RM66262 deploys the T2SS for both inter- and intraspecies competition.

### Complementation restores the wild-type phenotype, and the type II and VI secretion systems act synergistically

To confirm that the observed phenotype in the T2SS mutant strain is solely due to the absence of the structural protein GspD, a complemented strain was constructed. Additionally, to determine whether the simultaneous absence of both the T2SS and T6SS further impairs bacterial competition, a double mutant defective in both systems was generated.

Competition assays were performed using *E. coli* MC4100 as prey. The WT, the *gspD*, *tssM*, complemented (*gspD/*pGspD), and double mutant (*gspD tssM*) strains were used as attackers. The bacterial cultures were mixed at a 5:1 attacker-to-prey ratio, and colony-forming units (CFUs) were quantified by plating on LB–agar supplemented with streptomycin to select for the prey.

**Fig 4:**
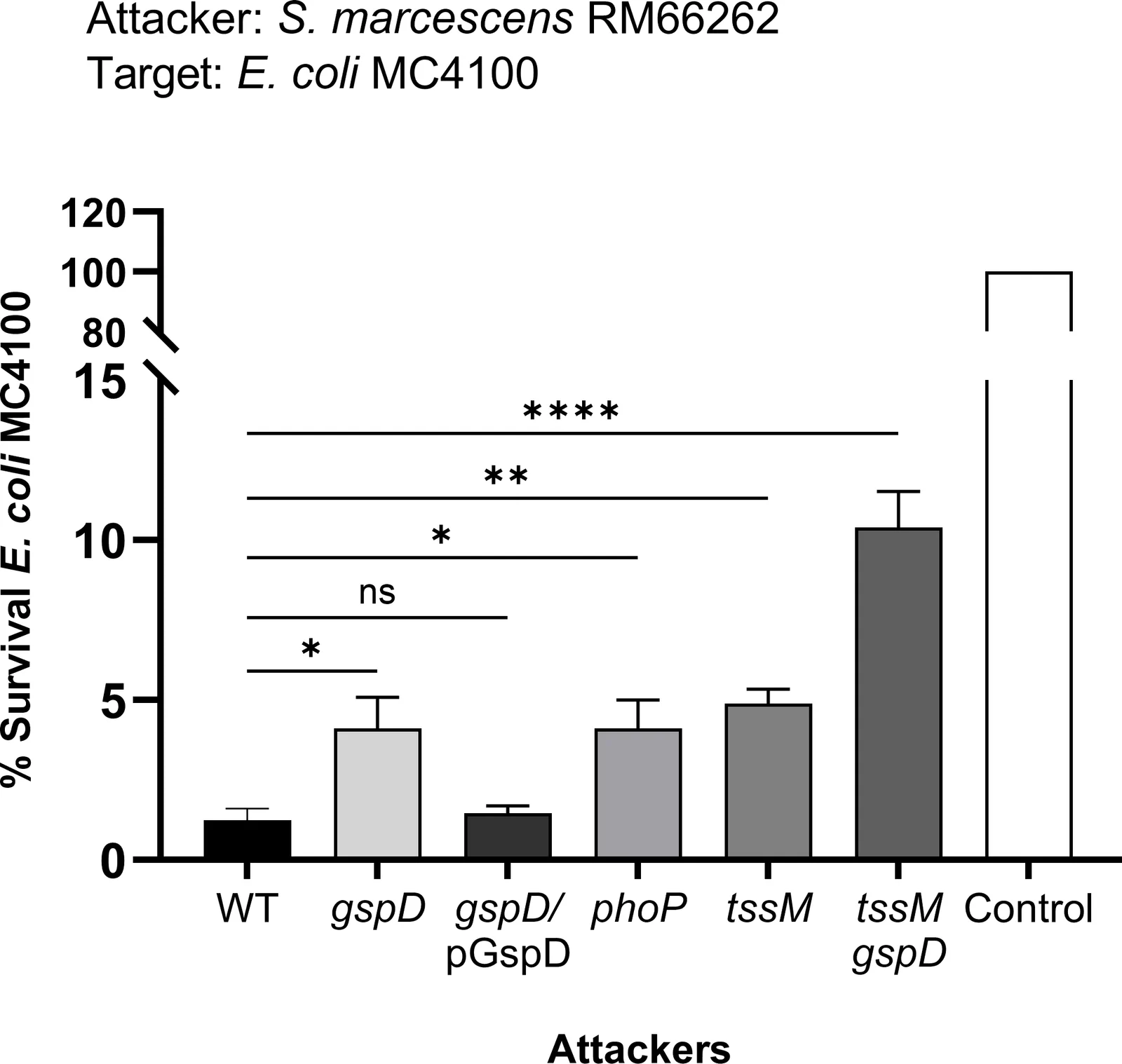
Analysis of T2SS function in interbacterial competition. Wild-type (WT), T2SS mutant (*gspD*), complemented mutant (*gspD*/pGspD), *phoP* mutant, T6SS mutant (*tssM*), and double mutant (*gspD tssM*) strains of *S. marcescens* RM66262 were used as attackers, while *E. coli* MC4100 served as the prey strain. Attacker and prey cells were co-incubated at a 5:1 ratio (attacker:prey) at 37°C for 4 h. *E. coli* DH5α mixed with *E. coli* MC4100 at a 5:1 ratio was used as a non-competition control. Percent survival was calculated as the ratio of prey CFU recovered from each competition assay to the CFU recovered from the non-competition control. Means ± SD of three independent experiments performed in duplicate in each case are shown. Statistical analysis was performed using one-way ANOVA followed by Dunnett’s multiple-comparison test. *, *P* < 0.05; **, *P* < 0.01; ****, *P* < 0.0001; ns, not significant.

The results showed that the complemented mutant restored the WT phenotype (Fig. 4). Moreover, the double mutant showed an approximately 10-fold decrease in competitive ability against *E. coli* MC4100 compared to either single mutant, suggesting a synergistic contribution of both secretion systems to interbacterial antagonism (Fig. 4).

### Identification of a putative PhoP-binding site in the T2SS

**Fig 5:**
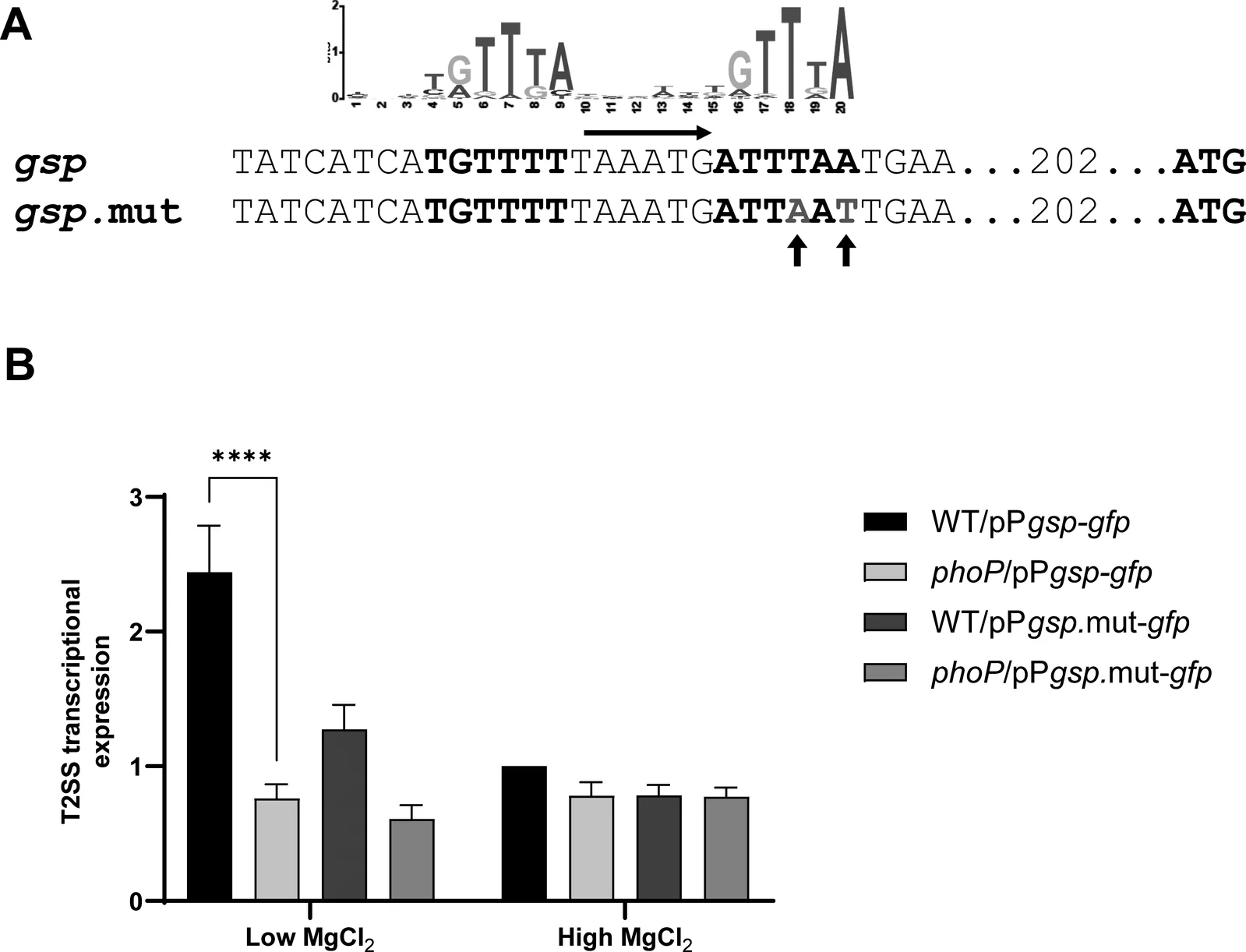
(**A**) PhoP-binding site within the putative regulatory region of the T2SS gene cluster in the *S. marcescens* RM66262 genome. A consensus motif for the PhoP-binding site was generated using a database of previously defined promoter regions of PhoP-regulated genes in *Salmonella enterica* serovar Typhimurium*, E. coli,* and *S. marcescens* and the MEME software tool. The logo (obtained using WebLogo [weblogo.berkeley.edu]) shows the consensus motif for the PhoP-binding site, and the putative PhoP-binding site sequences with high scores identified in the *S. marcescens* RM66262 genome by MAST upstream of the first gene of the T2SS cluster (*gsp*) are shown at the bottom. The PhoP boxes are shown in boldface; arrows indicate the mutated bases of the PhoP box in *gsp*.mut. The distance (in base pairs) from the translational start site is also indicated. (**B**) Transcriptional expression of the T2SS promoter and the T2SS promoter with two-point changes in the PhoP motif. Bacteria were grown for 16 h in N medium with 10 µM MgCl_2_ for low-Mg^2+^ conditions or 10 mM MgCl_2_ for high-Mg^2+^ conditions, in 96-well microplates, at 37°C with shaking. Transcriptional activity was calculated as the ratio of GFP fluorescence to OD₆₀₀, measured in strains carrying either the *pPgsp-gfp* or the *pPgsp*.mut*-gfp* reporter plasmids, and expressed relative to the wild-type strain under high-Mg^2+^ conditions. Means ± SD of three independent experiments performed in triplicate in each case are shown. Statistical analysis was performed using two-way ANOVA followed by Tukey’s multiple-comparison test. ****, *P* < 0.0001; ns, not significant.

Using multiple expectation-maximization for motif elicitation (MEME)/Motif Alignment and Search Tool (MAST) motif prediction algorithms, a putative PhoP-binding DNA motif was identified in the promoter region of the gene cluster encoding the T2SS components in *S. marcescens* RM66262. First, a PhoP-binding consensus motif was generated using MEME based on a set of promoter regions containing validated PhoP recognition sequences (32–35). Subsequently, a putative PhoP-binding site was identified 202 bp upstream of the ATG (start codon) of the first gene of the *S. marcescens* RM66262 T2SS operon using the MAST algorithm. Figure 5A illustrates the PhoP-recognition DNA sequence logo and the predicted PhoP recognition sites in the promoter region.

### PhoP-dependent regulation of T2SS expression in *Serratia marcescens*

To determine whether the T2SS is regulated by PhoP, we analyzed promoter activity using a *gfp* reporter gene. The transcriptional reporter plasmid pP*gsp-gfp*, carrying a *gfp* fusion to the 446-bp region upstream of the translational start codon of the first gene in the cluster (P*gsp*), was conjugated into both the WT and *phoP* mutant strains.

Fluorescence was measured after overnight growth at 37°C in N medium under inducing (10 μM Mg^2+^) and repressing (10 mM Mg^2+^) conditions for the PhoP/PhoQ system (36).

Consistent with our *in silico* prediction of a PhoP-binding motif, we found that P*gsp* transcription is differentially regulated at low- and high-Mg^2+^ concentrations. As shown in Fig. 5B, P*gsp* transcriptional levels were twofold higher at low Mg^2+^ in the wild-type strain compared to the *phoP* mutant. These results indicate that PhoP is involved in the regulation of T2SS expression.

To assess whether this regulation is mediated through direct interaction with the identified motif, we introduced two-point mutations within the T2SS promoter region in the reporter plasmid pP*gsp*-gfp via site-directed mutagenesis. Specifically, two highly conserved positions within the PhoP-binding motif of the T2SS promoter were substituted with their complementary nucleotides (Fig. 5A). The resulting plasmid, pP*gsp*.mut-*gfp*—carrying the mutated promoter (P*gsp*.mut)—was then conjugated into both WT and *phoP* mutant strains.

As shown in Fig. 5B, P*gsp*.mut-*gfp* expression was reduced by approximately 50% in the wild-type strain under low-Mg^2+^ conditions, indicating that PhoP does not efficiently recognize the mutated motif in the T2SS promoter. This result further supports the role of PhoP in the direct regulation of T2SS expression.

Altogether, these findings demonstrate that PhoP regulates the transcriptional activation of the T2SS gene cluster.

### Electrophoretic mobility shift assay confirms direct binding of PhoP to T2SS promoter regions

To confirm that PhoP directly regulates the T2SS, an electrophoretic mobility shift assay (EMSA) was performed. The PhoP-6xHis protein was purified, and the promoter regions of interest were amplified by PCR for use as DNA probes. The probes included the promoter region of the T2SS operon (P*gsp*), the mutated promoter (P*gsp*.mut), the *phoPQ* operon promoter (P*phoP*) as a positive control—since the *phoPQ* operon is autoregulated (34, 35)—and a fragment corresponding to the *nucA* gene as a negative control.

**Fig 6:**
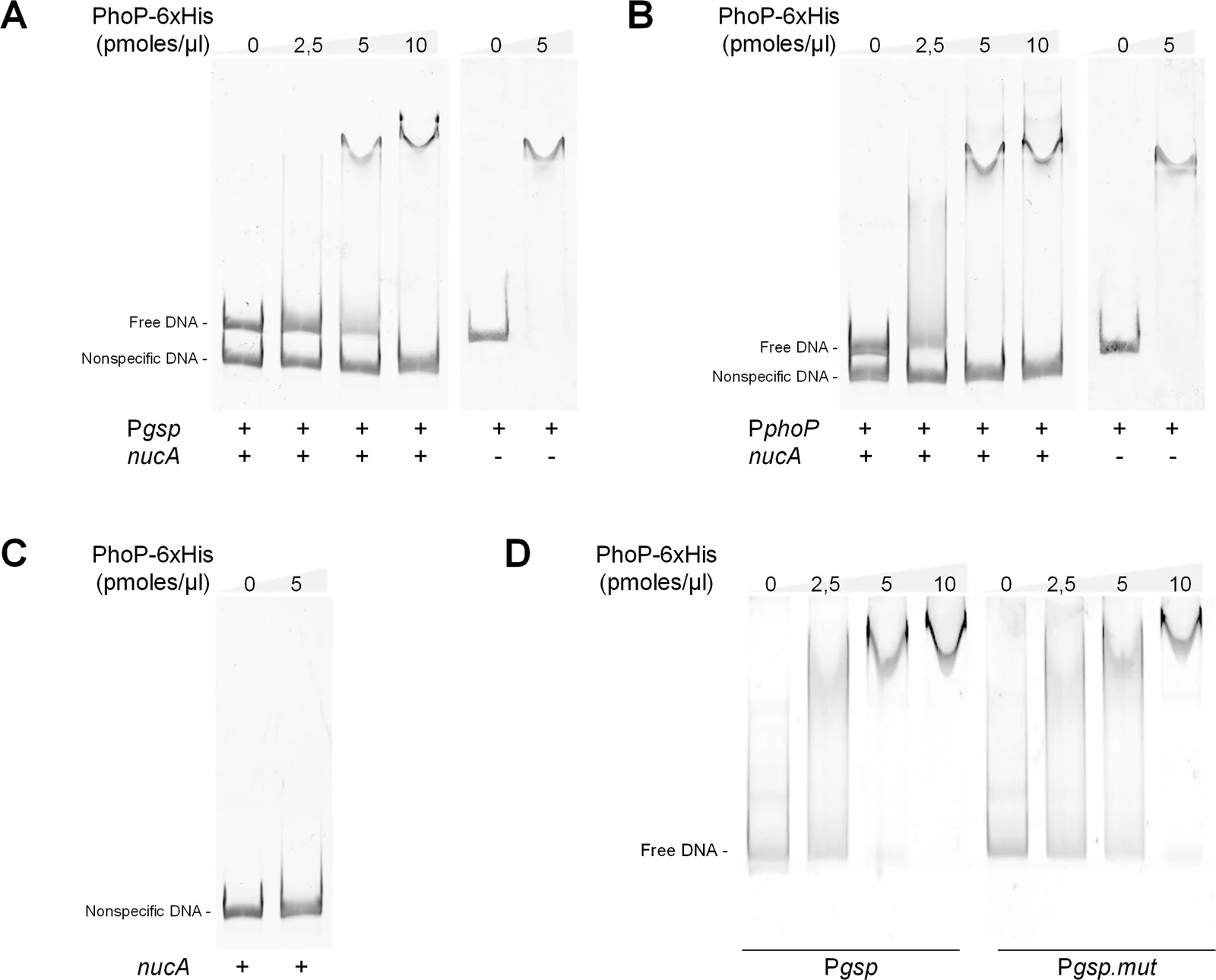
PhoP interaction with the promoter region of the T2SS cluster of *S. marcescen*s. Electrophoretic mobility shift assays (EMSAs) were performed using different amounts of purified PhoP-6xHis. The target DNA was a PCR fragment that included the promoter region of the T2SS operon (P*gsp*), the promoter region of the T2SS operon with two-point mutations (P*gsp*.mut), the promoter region of the *phoPQ* operon used as a positive control (P*phoP*), or a fragment corresponding to the *nucA* gene used as a negative control. Binding specificity was evaluated by competition reactions using 0, 2.5, 5, and 10 pmol of purified PhoP-6xHis and 35 ng of each DNA as a probe. (**A**) P*gsp* and *nucA* probes. (**B**) P*phoP* and *nucA* probes. (**C**) *nucA* probe with 0 or 5 pmol PhoP-6xHis. (**D**) P*gsp* and P*gsp*.mut probes.

When DNA fragments containing the promoter sequences of P*gsp* and P*phoP* were incubated with PhoP, shifted bands were detected whose intensities increased with higher concentrations of PhoP. This was accompanied by a decrease in the intensity of the unbound probe band (Fig. 6A and B). Moreover, PhoP did not bind to the non-specific DNA fragment (*nucA*) (Fig. 6C), and the presence of *nucA* did not interfere with the binding of PhoP to the P*gsp* and P*phoP* promoter sequences, as evidenced by the consistent intensity of the shifted bands despite increasing PhoP concentrations (Fig. 6A and B). As shown in Fig. 6D, increasing concentrations of PhoP induced a weaker mobility shift of the mutated P*gsp*.mut probe compared to the wild-type P*gsp* probe, accompanied by a higher proportion of unbound probe. This reduced shift likely reflects a lower affinity of PhoP for the mutated binding motif rather than a complete loss of binding, consistent with our reporter assays showing that PhoP-dependent regulation is diminished but not entirely abolished in the P*gsp*.mut construct. These results demonstrate that, as predicted, PhoP directly interacts with the promoter region of the T2SS operon through a specific PhoP-binding motif.

### Induction of T2SS during interaction with *Acinetobacter nosocomialis*

Expression of the T6SS is triggered when *S. marcescens* is exposed to an antagonistic species, such as *A. nosocomialis* M2 (28). Given the involvement of the T2SS in bacterial competition, we investigated whether its expression is also induced during interaction with a competing bacterium. *A. nosocomialis* M2 was selected because it is a well-characterized competitor of *S. marcescens* and its T6SS has previously been shown to elicit a T6SS-dependent response in RM66262 (28). To test whether T2SS expression is induced during this interaction, competition assays were performed using wild-type *A. nosocomialis* M2 as the attacker and an otherwise isogenic *tssD* mutant, which lacks a functional T6SS, as a control. The WT *S. marcescens* and the *phoP* mutant strains, both carrying the T2SS reporter plasmid (P*gsp*-gfp), were used as prey.

**Fig 7:**
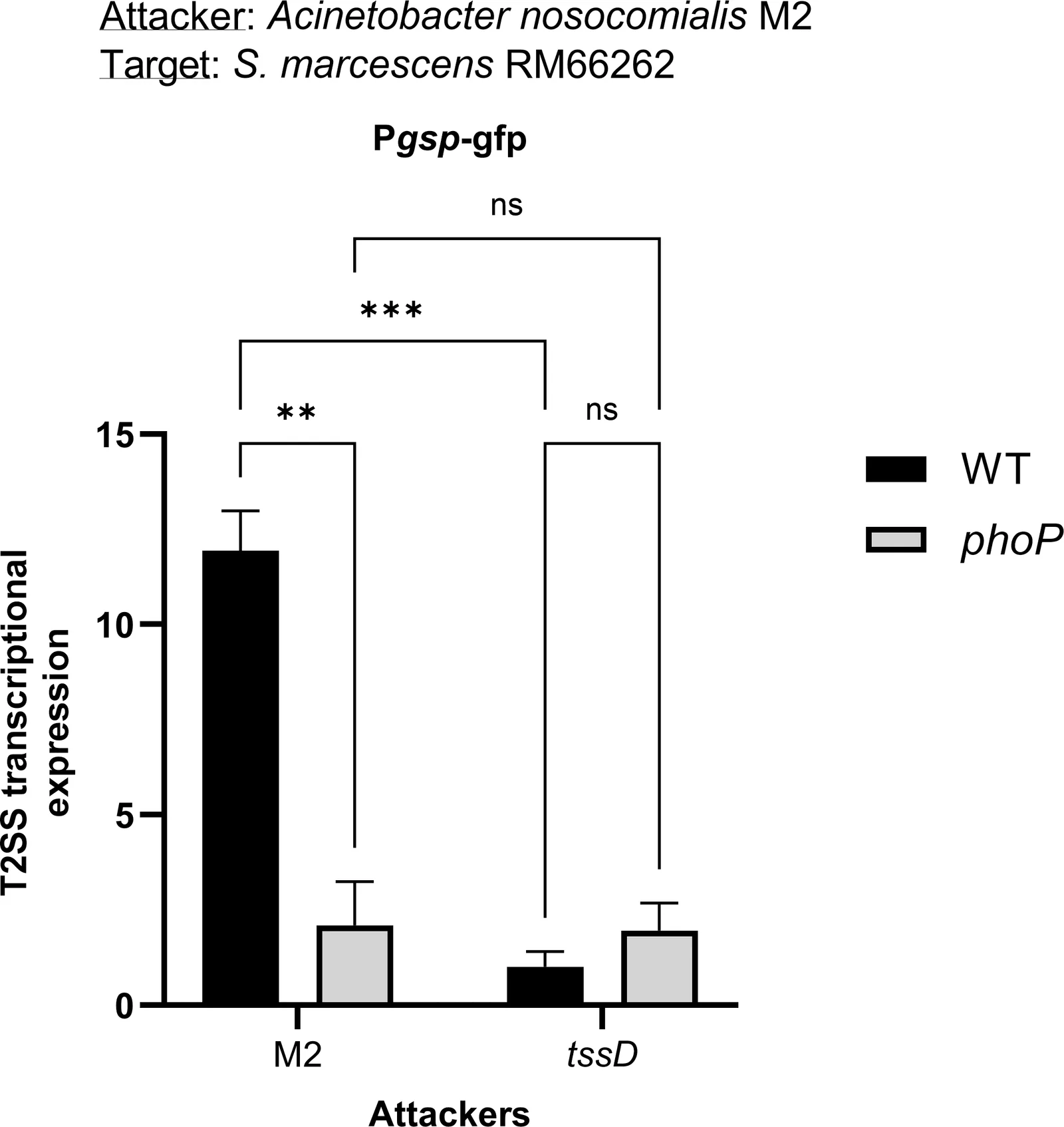
T2SS transcriptional induction by interspecific competition between *A. nosocomialis* and *S. marcescens. A. nosocomialis* (attacker) and *S. marcescens* RM66262 (prey) strains were co-incubated in a 5:1 ratio (attacker:prey) at 37°C for 4 h. The prey strains contained the transcriptional reporter plasmid pP*gsp-gfp*. The CFU of the prey strain was determined, and the GFP fluorescence intensity, expressed as fluorescence units (FU), was measured to obtain the FU/CFU ratio. Means ± SD of two independent experiments performed in triplicate in each case are shown. Statistical analysis was performed using two-way ANOVA followed by Tukey’s multiple-comparison test. **, *P* < 0.01; ***, *P* < 0.001; ns, not significant.

The prey strains were selected on LB–agar plates supplemented with ampicillin and spectinomycin. After co-incubation, cells were resuspended in PBS, GFP fluorescence was measured, and CFU counts were determined. T2SS transcription increased when *S. marcescens* was co-incubated with wild-type *A. nosocomialis* M2 but not with the otherwise isogenic *tssD* mutant, indicating that induction requires a functional T6SS in the competing bacterium (Fig. 7). This induction was not observed in the *phoP* mutant, indicating that PhoP is required for T2SS activation during this interaction. However, these experiments do not distinguish whether T2SS induction is triggered by T6SS-mediated intoxication or envelope damage, physical contact with an actively assembled T6SS, or another consequence of the interaction. Thus, although a functional T6SS in *A. nosocomialis* is required for T2SS induction under these conditions, the nature of the inducing signal sensed by PhoP/PhoQ remains unknown.

### PhoP is required for T2SS-dependent predation

Since PhoP/PhoQ was shown to regulate T2SS expression, we performed competition assays to determine whether the *phoP* mutant exhibits an attenuated killing phenotype similar to that of the T2SS mutant. These assays were conducted at a 5:1 attacker-to-prey ratio, using *S. marcescens* WT, *gspD* mutant, and *phoP* mutant as attackers *and E. coli* MC4100 as prey. Under PhoP/PhoQ-inducing conditions (i.e., low-Mg²^+^ medium LB), the *phoP* mutant lost the ability to kill the prey strain, similar to the *gspD* mutant. This result further supports the conclusion that PhoP/PhoQ regulation of the T2SS contributes to the competitive ability of *S. marcescens* (Fig. 4).

## DISCUSSION

This study reveals a previously unrecognized function of the type II secretion system (T2SS) in *Serratia marcescens*, specifically in mediating bacterial competition. While the T2SS has been well characterized in various gram-negative pathogens for its role in secreting extracellular virulence factors such as proteases, lipases, and toxins (16–21), its involvement in interbacterial antagonism had not been described, nor had its association with clinical *Serratia* isolates.

Comparative genomic analysis between the clinical isolate RM66262 and the environmental reference strain Db11 revealed the absence of the T2SS in the latter. A broader phylogenetic survey demonstrated that the T2SS is conserved exclusively among clinical isolates, suggesting its acquisition or positive selection in highly competitive environments, such as hospital settings or the human host. This distribution implies an adaptive advantage conferred by T2SS-mediated antagonism in polymicrobial contexts.

Consistent with this hypothesis, functional assays demonstrated that the T2SS in *S. marcescens* contributes to bacterial killing, targeting both heterologous species (*Escherichia coli*, *Pseudomonas aeruginosa*) and an isogenic strain (Db11). Deletion of the T2SS secretin gene (*gspD*) abolished this activity, phenocopying the *tssM* mutant, which lacks a functional T6SS. Importantly, our results indicate that the T2SS mutant retains a functional T6SS, as evidenced by the continued secretion of Hcp, a hallmark of T6SS activity (37). Notably, the T6SS had previously been the only secretion system associated with bacterial antagonism in *S. marcescens* (28, 37). Together, these results demonstrate that both the T2SS and T6SS contribute to competitive interactions. In line with this model, a recent study reported that a clinical strain belonging to the same lineage as ours exhibits reduced T6SS activity (38). Notably, we detected the presence of the T2SS in this strain, suggesting that these secretion systems may be differentially deployed across hospital-adapted lineages and that different clinical lineages may rely to different extents on these secretion systems depending on their ecological or host-associated context.

The exacerbated defect observed in the *gspD tssM* double mutant is consistent with the T2SS and T6SS contributing through distinct yet complementary mechanisms to promote competitive fitness. While the T6SS mediates contact-dependent killing via direct injection of toxic effectors into neighboring cells, the T2SS secretes extracellular enzymes and other protein substrates that may contribute to bacterial competition. These findings suggest that the T2SS and T6SS mediate interbacterial antagonism through complementary mechanisms, although the specific contribution of T2SS-secreted factors remains to be elucidated. Understanding how the T2SS and T6SS are coordinated will be essential to fully elucidate their roles in complex microbial communities. Interestingly, the residual competitive activity observed in the *gspD tssM* double mutant suggests that additional antibacterial mechanisms may also contribute to bacterial competition in *S. marcescens*. Identifying these factors and determining how they integrate with the T2SS and T6SS will require further investigation.

Importantly, the competitive defect of the *gspD* mutant was rescued by complementation, confirming that the observed phenotype is directly attributable to the loss of T2SS function.

At the regulatory level, we identified the PhoP/PhoQ two-component system as a direct activator of T2SS expression. PhoP activates transcription of the T2SS operon through direct binding to a conserved motif within its promoter region. This expands our knowledge of the PhoP regulon in *S. marcescens*, which has previously been associated primarily with outer membrane remodeling, intracellular trafficking, and resistance to antimicrobial peptides (34, 35). The presence of PhoP-binding sites in the promoter regions, validated by EMSA and reporter assays, supports direct transcriptional control.

Interestingly, our results reveal a clear regulatory distinction between these two secretion systems. The T2SS is directly regulated by the PhoP/PhoQ system and not by the Rcs phosphorelay (See Fig. S2 at https://ibr-conicet.gov.ar/wp-content/uploads/2026/09/Spectrum-Sartori-et-al-Supplemental-Material.pdf); moreover, no putative RcsB-binding site was identified in the *gsp* promoter region. In contrast, our previous studies demonstrated that the T6SS in *S. marcescens* is regulated by the Rcs phosphorelay and is not controlled by PhoP/PhoQ (28). Consistent with this, Hcp was detected in the secretome of the *phoP* mutant (Fig. 3D), indicating that the T6SS remains functional in the absence of PhoP and confirming that T6SS activity is independent of PhoP/PhoQ regulation. This divergence in regulatory control indicates that the T2SS and T6SS are controlled by distinct regulatory pathways. Whether these pathways respond to different environmental cues or to different consequences of the same T6SS-mediated attack remains to be determined. Such regulatory segregation may allow *S. marcescens* to differentially regulate these secretion systems during interbacterial competition. This distinct regulatory control is consistent with the complementary roles of the T2SS and T6SS in bacterial competition.

We further demonstrate that T2SS expression is induced during interaction with *Acinetobacter nosocomialis* carrying a functional T6SS and that this response is PhoP dependent. These findings reveal that T2SS expression is not constitutive but can be upregulated during interbacterial competition. Although the presence of a functional T6SS in *A. nosocomialis* is required for this induction, our experiments do not establish whether the inducing signal involves direct cell-to-cell contact, T6SS-mediated envelope damage, or another consequence of the interaction. In particular, we cannot determine from our data whether PhoP/PhoQ is activated directly in response to membrane or envelope damage. Identifying the signal sensed by PhoP/PhoQ during this interaction will require further investigation. Although the T6SS has traditionally been considered a contact-dependent secretion system, recent evidence indicates that some T6SS effectors can mediate antibacterial activity in the absence of direct cell-to-cell contact (39). This inducible regulatory pattern expands the functional repertoire of the T2SS beyond its classical roles in extracellular protein secretion and host-associated virulence, suggesting that it may also contribute to adaptive responses during bacterial competition.

This finding may be particularly relevant in polymicrobial healthcare-associated infections, where *S. marcescens* can coexist with other opportunistic gram-negative pathogens, including *Pseudomonas aeruginosa*, *Acinetobacter* spp., *Klebsiella pneumoniae*, and *Enterobacter* spp. (40). In these environments, polymicrobial communities are frequently exposed to antibiotic pressure, which may alter competitive interactions and impose additional selective pressures on bacterial populations. Under such conditions, secretion system-mediated antagonism, together with antimicrobial resistance, could contribute to the persistence and ecological success of particular clinical lineages and potentially influence the maintenance or displacement of multidrug-resistant populations within polymicrobial communities. However, the interplay between antibiotic pressure, antimicrobial resistance, and secretion system-mediated competition was not addressed in the present study and remains to be investigated.

Although the specific substrates secreted by the T2SS in *S. marcescens* RM66262 remain unidentified, genome analysis revealed several genes encoding proteins with predicted extracellular functions, including putative proteases, chitinases, lipases, and peptidoglycan hydrolases. These proteins are typical T2SS substrates in other gram-negative bacteria and may contribute to nutrient acquisition, tissue colonization, or interbacterial antagonism (16, 22). Further studies are required to experimentally identify these effectors and determine their role in T2SS-mediated competition.

The observation that the T2SS is conserved among clinical isolates but largely absent from environmental strains is consistent with a role for this secretion system in host-associated or hospital-relevant environments, where microbial competition and immune pressure are intense. However, the prevalence of the T2SS among clinical isolates could also reflect lineage-specific inheritance, and our data do not distinguish between these evolutionary scenarios. In hospital-associated environments, where polymicrobial communities are frequently exposed to antimicrobial treatment, competitive mechanisms such as the T2SS and T6SS may influence not only bacterial survival but also community composition. It is conceivable that, together with antimicrobial resistance determinants, secretion systems contribute to the persistence and competitive success of particular clinical lineages. Although this possibility was not addressed in the present study, it highlights the potential ecological relevance of secretion systems in healthcare-associated infections and warrants further investigation. Given its role in bacterial antagonism and its likely involvement in host colonization, the T2SS nevertheless emerges as a potential target for the development of novel therapeutic strategies aimed at disarming opportunistic pathogens without relying on conventional antibiotics. Together, our data establish that the T2SS of *S. marcescens* RM66262 contributes to both intra- and interspecies bacterial competition, and that its expression is tightly regulated by PhoP under competitive stress. These findings broaden the ecological and functional significance of the T2SS and underscore its potential relevance in polymicrobial infections and dysbiotic environments.

## MATERIALS AND METHODS

### Bacterial strains and plasmids

*Serratia marcescens* RM66262 is a nonpigmented clinical isolate from a patient with a urinary tract infection (GenBank accession no. NZ_JWLO00000000.1) (11). The bacterial strains and plasmids used in this study are listed in Table S1 (See at https://ibr-conicet.gov.ar/wp-content/uploads/2026/09/Spectrum-Sartori-et-al-Supplemental-Material.pdf). Bacteria were routinely grown in Miller’s LB medium or on LB agar plates overnight at 37°C. To assay Mg^2+^ regulation, strains were grown overnight under low-Mg^2+^ conditions {N medium [0.1 M Tris-HCl (pH 7.4), 0.1% (wt/vol) Casamino Acids, 38 mM glycerol, 0.37 g/L of KCl, 0.99 g/L of (NH_4_)_2_SO_4_, 0.087 g/L of K_2_SO_4_, 0.14 g/L of KH_2_PO_4_] (41) plus 10 µM MgCl_2_} or high-Mg^2+^ conditions (N medium plus 10 mM MgCl_2_). The antibiotics used were ampicillin (100 µg/mL), kanamycin (50 µg/mL), streptomycin (100 µg/mL), and gentamicin (15 µg/mL).

*S. marcescens gspD* and *tssM* deletion mutants were generated by allelic exchange using the suicide vector pKNG101 (42). Briefly, DNA fragments of approximately 500 bp corresponding to the regions upstream and downstream of each target gene were amplified by PCR using the primers listed in Table S2 (See at https://ibr-conicet.gov.ar/wp-content/uploads/2026/09/Spectrum-Sartori-et-al-Supplemental-Material.pdf) and fused by splice overlap extension (SOE)-PCR to generate deletion alleles of approximately 1 kb. The resulting constructs were cloned into the BamHI and SpeI sites of pKNG101 and introduced into *S. marcescens* RM66262 by conjugation. Mutant strains were selected on streptomycin, and double-crossover recombinants were isolated by sucrose counterselection. Gene deletions were confirmed by PCR. The *gspD tssM* double mutant was generated by introducing the *tssM* deletion into the *gspD* background using the same procedure.

To analyze the transcriptional levels of the T2SS cluster (*gsp*)*,* the promoter region was amplified by PCR using the primers prom.*gsp*-fw and prom.*gsp*-rv (See Table S2 at https://ibr-conicet.gov.ar/wp-content/uploads/2026/09/Spectrum-Sartori-et-al-Supplemental-Material.pdf). The purified PCR products were digested with the HindIII and XbaI restriction enzymes and were ligated into the same sites of pPROBE-OT (43). The resulting plasmid was introduced into competent *E. coli* TOP10 cells by transformation. The plasmid pP*gsp-gfp* was mobilized by conjugation into the *S. marcescens* wild-type and the *phoP* mutant strains.

For complementation of the S. marcescens *gspD* mutant strain, the *gspD* gene was amplified from the *S. marcescens* wild-type strain’s genomic DNA by PCR using primers gspD-Fw.BamHI and gspD-Rv.HindIII (See Table S2 at https://ibr-conicet.gov.ar/wp-content/uploads/2026/09/Spectrum-Sartori-et-al-Supplemental-Material.pdf). The PCR product was cloned into the pSU36 plasmid (44). The construct was then introduced into the *S. marcescens* strains by conjugation.

### Phylogenetic analysis

The 21 complete genome sequences of *S. marcescens* were retrieved from the National Center for Biotechnology Information (NCBI) Genome website (https://www.ncbi.nlm.nih.gov/datasets/genome/) as of 11 October 2023. The phylogenetic tree based on the whole-genome alignments was constructed using REALPHY 1.13 (25).

### Antibacterial competition assays

Competition assays were performed as previously described (28), with the following modifications. Bacterial cells grown overnight were normalized to an optical density at 600 nm (OD_600_) of 0.5 and mixed at a 5:1 or 10:1 (attacker:prey) ratio, as indicated in each figure legend. Attacker-to-prey ratios were selected based on the assay described by Lazzaro et al. and adjusted according to the susceptibility of the target strain, with more resistant prey requiring a higher attacker-to-prey ratio to achieve reproducible competition outcomes (28). A total of 25 µL of the mixture was spotted onto prewarmed agar plates and incubated at 37°C for 4 h. Cells were then recovered from the spots and resuspended in 1 mL of LB broth. Serial dilutions were plated on selective media containing streptomycin for *E. coli* strain MC4100 and *S. marcescens* strain Db11, or kanamycin for *Pseudomonas aeruginosa* PAO1. A mixture of *E. coli* DH5α and *E. coli* MC4100 at the same ratio was used as a non-competition control. Percent survival was calculated as the ratio of prey CFU recovered from each competition assay to the CFU recovered from the non-competition control. To assess whether differences in competitive ability were associated with general growth defects, CFU counts of the WT, *gspD*, *tssM*, *phoP*, and *gspD tssM* attacker strains were also determined under the same conditions used for the competition assays. Results represent the average of duplicate measurements from at least three independent experiments.

### Determination of secreted Hcp protein levels by immunodetection

Protein levels were determined by immunodetection as described in reference 45. Briefly, 30 mL of cultures were grown overnight with good aeration in LB at 37°C and normalized by OD600. Cultures were centrifuged for 5 min at 5,000 × *g*, and the supernatant was removed. This was filtered through 0.2 μm acetate-cellulose filters, precipitated with 12% trichloroacetic acid for 2 h at 4°C, and centrifuged for 30 min at 30,000 × *g*. The precipitated secreted proteins were resuspended in a protein sample buffer. A total of 20 ng of proteins were run in parallel on two 18% sodium dodecyl sulfate polyacrylamide electrophoresis (SDS-PAGE) gels, and one of these was transferred to Hybond-ECL nitrocellulose membranes. The membrane was blocked for 1 h with 5% skimmed milk and Tris-buffered saline (TBS: 20 mM Tris, 150 mM NaCl, pH 7.6) and washed three times with TBS for 10 min. The membrane was then incubated with rabbit anti-*S*. *marcescens* Hcp polyclonal antibodies, washed three times with TBS, and finally incubated with an horseradish peroxidase-conjugated secondary antibody. The membrane was developed using an enhanced chemiluminescence detection kit (Thermo Fisher Scientific). The twin gel was stained with Coomassie blue for 1 h. The assay was repeated twice.

### Bioinformatics analysis

To search for the putative PhoP-binding sites in the T2SS promoter region, the MEME Suite were used, a collection of tools for the discovery and analysis of sequence motifs. In brief, MEME is an algorithm that, given multiple input sequences, identifies one or more candidate motifs. MEME outputs the motif with the lowest (estimated) E value. Then, MAST searches for the motif in sequence databases using the position-specific probability matrix created by MEME (46, 47).

### Site-directed mutagenesis by QuickChange PCR

This technique was employed to introduce site-directed mutations in the putative PhoP-binding box within the promoter of the T2SS operon, carried on the reporter plasmid pPROBE::P*gsp-gfp*. The oligonucleotides prom.gsp.mut-F and prom.gsp.mut-R were designed to anneal within the predicted PhoP-binding site and contained the point mutations indicated in Fig. 5. The reaction was performed following the manufacturer’s protocol for Q5 High-Fidelity DNA Polymerase (New England Biolabs). The thermal cycling program consisted of an initial denaturation at 98°C for 30 s, followed by 25 cycles of denaturation at 98°C for 15 s, primer annealing at 56°C for 30 s, and DNA extension at 72°C for 5 min. A final extension step was included at 72°C for 3 min. After amplification, 1 μL of DpnI enzyme was added to the PCR product, and the mixture was incubated at 37°C for 3 h to digest the methylated parental DNA. The reaction was then heat-inactivated at 65°C for 10 min. The digested product was transformed into *E. coli* TOP10 cells to recover colonies carrying the mutated plasmid, which were subsequently confirmed by sequencing. Finally, the plasmid pPROBE::P*gsp*.mut-*gfp* was conjugated into both the wild-type and *phoP* mutant strains of *Serratia*.

### GFP transcriptional reporter assay

Cultures of wild-type (WT) and *phoP* mutant *S. marcescens* strains carrying the pP*gsp-gfp* and pP*gsp*.mut*-gfp* reporter plasmids were grown with shaking overnight at 37°C. The bacterial cultures were washed twice with N medium; 50 µL was mixed with 950 µL of N medium supplemented with 10 µM or 10 mM MgCl_2_ with spectinomycin, and 100 µL volumes of the mixtures were incubated in a 96-well microtiter plate at 37°C with agitation for 16 h. Optical density at 600 nm (OD600) and GFP fluorescence (excitation wavelength [*λ*ₑₓc] of 485 nm and emission wavelength [*λ*ₑₘ] of 528 nm) were determined using a 96-microwell plate reader (Synergy 2). Transcriptional activity was calculated as the ratio of GFP fluorescence to OD₆₀₀, measured in strains carrying the *pPgsp-gfp* and *pPgsp*.mut*-gfp* reporter plasmids, and expressed relative to the wild-type strain under high-magnesium conditions. The means and standard deviations for three independent assays performed in triplicate in each case were calculated.

### Protein-DNA interaction analysis

Electrophoretic mobility shift assays (EMSAs) were performed using DNA probes from the promoter regions of the T2SS operon (P*gsp*) and from the *gsp.mut* (P*gsp*.mut) and *phoP* (P*phoP*) genes (positive control), with different amounts of purified PhoP-6xHis protein, essentially following previously described protocols (28, 35, 45, 48). The specificity of the binding was tested by co-incubation of the P*gsp* and P*phoP* probes with a 441 bp PCR fragment corresponding to the *nucA* gene of *S. marcescens* as a non-specific competitor. To evaluate the interaction of PhoP with the promoter region of the T2SS cluster, EMSAs were performed using 35 ng of DNA fragments containing the promoter regions of the genes and 0, 2.5, 5, and 10 pmol of purified PhoP-6xHis. The primers used to amplify the P*gsp,* P*gsp*.mut, and P*phoP* regions and *nucA* are listed in Table S2 (See at https://ibr-conicet.gov.ar/wp-content/uploads/2026/09/Spectrum-Sartori-et-al-Supplemental-Material.pdf). After electrophoresis, the gels were stained with SYBR Green (Invitrogen). DNA and protein-DNA complexes were detected and captured using a Typhoon FLA7000 laser scanner (GE Healthcare).

### Antibacterial competition assay with GFP transcriptional reporter

The competition assays with a GFP transcriptional reporter were performed as described above, with modifications as follows. The target strain carried the P*gsp-gfp* construct. Serial dilutions were plated on media containing ampicillin and spectinomycin for selection of the reporter strain. Cells were washed three times with phosphate-buffered saline (PBS), and GFP fluorescence (excitation [*λ*ₑₓc] at 485 nm and emission [*λ*ₑₘ] at 528 nm) was determined using a 96-microwell plate reader (Synergy 2). Transcriptional induction was calculated as fluorescence divided by CFU. The results for each experiment are the average values of an assay performed in triplicate and independently repeated at least three times.

### Statistical analysis

Statistical analysis was performed using one-way or two-way analysis of variance (ANOVA), as appropriate. For Fig. 4, statistical significance was assessed using one-way ANOVA followed by Dunnett’s multiple-comparison test. For Fig. 5 and 7, statistical significance was assessed using two-way ANOVA followed by Tukey’s multiple-comparison test. Statistical significance was set at *P* < 0.05. When technical duplicates were included, the mean of the technical duplicates was calculated for each biological replicate and used as a single experimental value for statistical analysis. Each biological replicate represents an independent experiment performed using independently grown overnight cultures. Asterisks in the plots indicate statistically significant differences between the indicated groups.

## Data Availability

All data supporting the findings of this study are available within the article and its supplemental material files, or from the corresponding author upon reasonable request. No large-scale data sets were generated during this study. Supplemental tables and figures can be found at https://ibr-conicet.gov.ar/wp-content/uploads/2026/09/Spectrum-Sartori-et-al-Supplemental-Material.pdf.
